# Outcomes of Prostate Biopsy in Men with Hypogonadism Prior or During Testosterone Replacement Therapy

**DOI:** 10.1590/S1677-5538.IBJU.2014.0528

**Published:** 2015

**Authors:** Daniel A Shoskes, Yagil Barazani, Khaled Fareed, Edmund Sabanegh

**Affiliations:** 1 Department of Urology, Glickman Urological and Kidney Institute, The Cleveland Clinic, Cleveland, OH, USA

**Keywords:** Hypogonadism, Testosterone, Biopsy

## Abstract

**Introduction:**

The relationship between Testosterone Replacement Therapy (TRT) and prostate cancer remains controversial. Most TRT studies show no change in prostate specific antigen (PSA) but some men do have PSA rise or develop an abnormal digital rectal exam (aDRE). Our objective was to examine the biopsy results of men with symptomatic hypogonadism before or during therapy.

**Materials and Methods:**

Data was extracted from our medical record on men with hypogonadism who had a prostate biopsy within the past 4 years done by 3 Urologists with guideline driven practice patterns.

**Results:**

96 men were identified. Mean age at biopsy was 63 (range 40–85) and median PSA was 3.78ng/dL (0.5–662). Of the 61 men not on TRT, median PSA was 4.34 (0.5 to 662) and mean total testosterone 254 (191–341). There were 29 (47.5%) prostate cancers found (6 Gleason score 6, 13 Gleason score 7, 10 Gleason score 8 or 9). Of the 35 men on TRT, median PSA was 3.27 (0.5 to 13.7). The %PSA increase ranged from 2 to 251% (mean 93.5%). Mean total testosterone was 383 (146–792). Of the 14 men treated < 2 years, none had cancer. Of the 21 men treated 2 or more years 5 had cancer (2 Gleason score 6, 3 Gleason score 7).

**Conclusions:**

Men with hypogonadism and a clinical indication for biopsy often have prostate cancer, many high grade. No men with an initial PSA rise on TRT had cancer. Men on long term TRT should be monitored with PSA and DRE per guidelines.

## INTRODUCTION

The interaction between Testosterone Replacement Therapy (TRT), hypogonadism and prostate cancer remains controversial. While prostate cancer has remained a contraindication in the package insert of TRT formulations, an emerging body of evidence suggests that TRT does not increase the risk of developing prostate cancer (1), is safe following therapy for prostate cancer (2) and may be safe during active surveillance of prostate cancer (3). Current guidelines recommend measuring testosterone prior to starting TRT and age appropriate monitoring of Prostate Specific Antigen (PSA) and digital rectal exam before and during therapy (4). Anecdotal evidence so far in North America is that these guideline recommendations are often not followed (5).

In studies of TRT, mean PSA usually remains stable (6) or rises slightly (7). Nevertheless, individual patients may have a significant jump in PSA after starting TRT which is concerning for prostate cancer and often prompts a prostate biopsy. We have not found any published studies to date that address how often these patients are found to actually have prostate cancer in this clinical scenario.

## AIM

To study the results of prostate biopsy in men with a diagnosis of hypogonadism, comparing the outcomes both prior to and after starting TRT, with an emphasis on men whose PSA rapidly rises following initiation of therapy.

## MATERIALS AND METHODS

Under an Institutional Review Board approved protocol we retrospectively extracted data from the Cleveland Clinic electronic medical record of patients with a prostate biopsy done between 2010 to 2014 with a concurrent clinical diagnosis of hypogonadism (CPT 257.2). We focused on patients cared for by three urologists specialized in the evaluation and treatment of men with low testosterone and who all follow common clinical guidelines for diagnosis, treatment and monitoring (4). Initially 125 men were identified. Following individual chart review 29 men were excluded for reasons such as prior prostate cancer, incorrect diagnosis or missing data. This left 96 patients for analysis. Data collected included age, initial PSA, prior PSA, initial morning total testosterone, testosterone after therapy (most recent value prior to biopsy), duration of therapy and pathology of biopsy including Gleason score. Men on testosterone therapy received a variety of agents including injections, topical gels and implantable subcutaneous pellets. All patients had a minimum of 12 cores taken for pathology and graded according to the latest Gleason score. The study period predates our use of MRI-fusion biopsy.

Data was analyzed using Prism 5.0 for Mac (GraphPad). Continuous variables were compared by t test or ANOVA when parametric and Mann-Whitney or Kruskal-Wallis test when non-parametric. Categorical outcomes were compared by the Chi squared test with Fischer correction. All tests were double sided. Statistical significance was set at P<0.05.

## RESULTS

Overall 96 men were identified. Mean age at time of biopsy was 63±9 years (range 40–85) and median PSA was 3.78ng/dL (0.5–662). There were 61 men with low testosterone who had not yet started TRT who were biopsied before starting therapy due to elevated PSA or abnormal DRE. In this group mean age was 64 years, median PSA 4.34 (0.5 to 662) and mean total testosterone 276ng/dL (191–341). There were 9 men with PSA<2.5 whose indication for biopsy was an abnormal DRE. Of 61 biopsies in men pre TRT, 29 (47.5%) had prostate cancer. Of these cancers there were six Gleason score 6 tumors, thirteen Gleason score 7, five Gleason score 8 and five Gleason score 9. Two men had metastatic disease at presentation. There was no significant difference in testosterone between men with a normal biopsy and those with cancer (257±12.9 vs 250.0±14.9, p=0.72), whether measured in aggregate or separating Gleason score 7 or above (total testosterone 258.6±8.2). By contrast, the PSA was significantly lower in those with a normal biopsy vs cancer (median 3.4 vs 5.0, p<0.0001 by Mann-Whitney) driven by high PSA values in those with high grade disease (Gleason score 7 or higher median PSA 5.12, p=0.0002 by Kruskal-Wallis with Dunn Multiple Comparison test). For the 32 biopsies without cancer, 11 had prostatic intraepithelial neoplasia and 9 had some degree of parenchymal inflammation.

**Figure 1:**
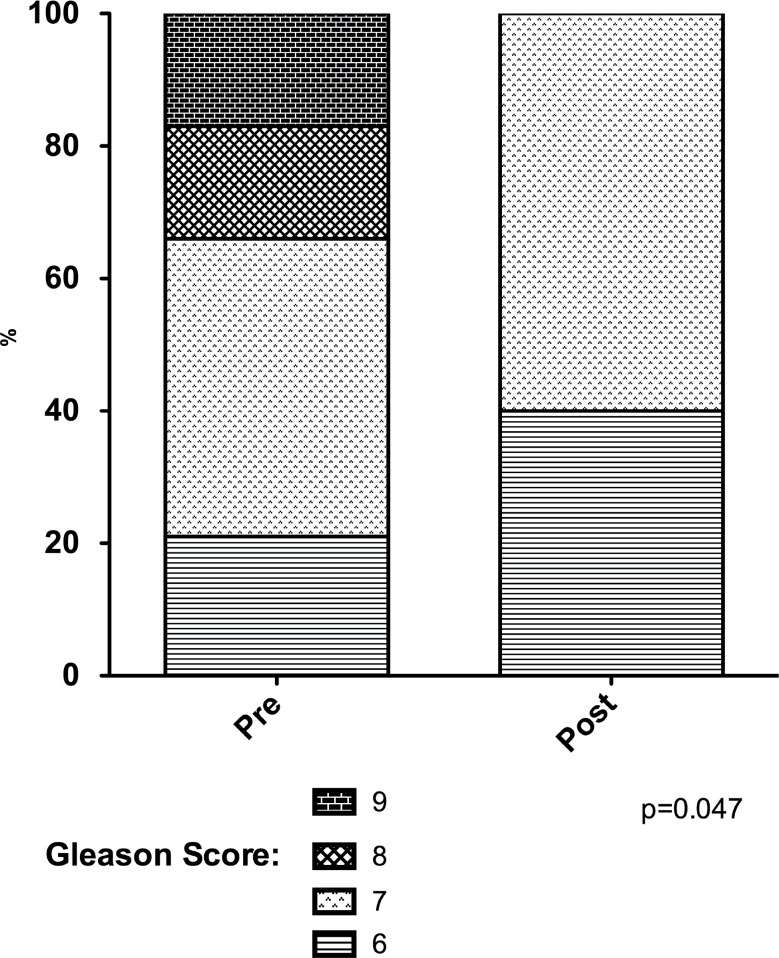
Comparison of Total Gleason Score Distribution in Patients Prior to or Receiving Testosterone Replacement Therapy.

**Figure 2:**
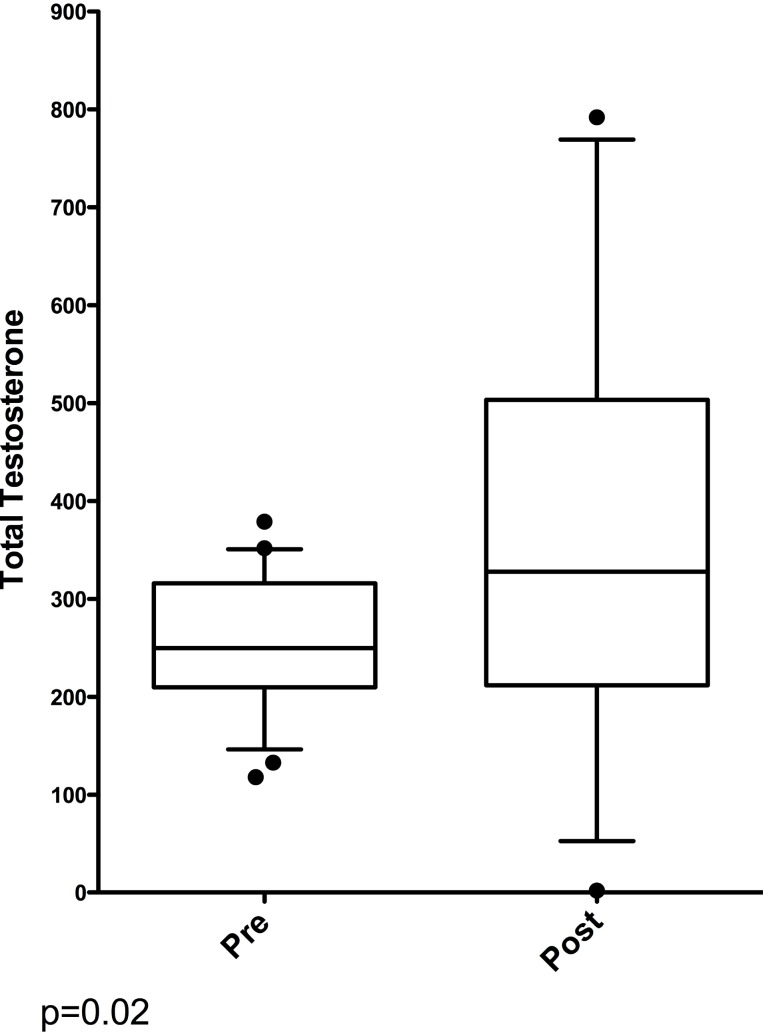
Box Plot of Testosterone Levels Before and After Testosterone Replacement Therapy.

Of the 35 men on TRT, mean age was 60 years and median PSA was 3.27ng/mL (range 0.5 to 13.7). Nine had PSA<2.5 but were biopsied due to an abnormal DRE. The % increase PSA compared to the prior value ranged from 2 to 251% (mean 93.5%). Mean total testosterone was 383ng/dL (146–792) which was significantly higher than the pre TRT group (P=0.047) (Figure-1). Of the 14 men treated for less than 2 years, none had cancer. Of the 21 men treated 2 or more years there were 5 cancers (two Gleason score 6 and three Gleason score 7) (Figure-2). There was no difference between those men on TRT with a normal biopsy vs cancer for testosterone (median 328 vs 388 p=0.58) or PSA (median 3.27 vs 2.76 p=0.81). For the 24 biopsies without cancer, 8 had prostatic intraepithelial neoplasia and 9 had some degree of parenchymal inflammation.

## DISCUSSION

Much current controversy surrounds TRT in men, particularly related to risk of development and progression of prostate cancer. While most prostate cancer cells require testosterone for growth, emerging evidence supports a testosterone “saturation hypothesis” which states that cells require a relatively low concentration of testosterone for maximal receptor engagement and higher exposure does not further promote growth (8). Indeed, some studies have implicated low serum testosterone as a risk factor for the development of prostate cancer, particularly high grade tumors (9). Furthermore, PSA levels either don't rise or rise modestly during long term TRT (6). Indeed, published guidelines do recommend screening men for prostate cancer prior to initiating TRT and then monitoring PSA and DRE during therapy (4). Surprisingly, in one study over half of men on TRT did not have a testosterone or PSA prior to starting therapy and did not have monitoring while on therapy (5).

The genesis of this study was an observation that some men starting TRT had an early sharp increase in PSA but that all biopsies done in response to this rise were negative. When collecting data from our Men's Health registry, we restricted the patients to the practice of three urologists of the staff with a specialty interest in TRT who closely follow treatment guidelines, specifically checking PSA and DRE in age appropriate men before and after therapy. Our findings have clinical implication for patients considering therapy and for those on treatment.

There were 61 men who had low T and had a prostate biopsy due to an abnormal DRE or elevated PSA during workup prior to starting TRT. There were 29 cancers and 10 were Gleeson 8 or 9. An association between low testosterone and high grade prostate cancer has previously been reported (10). Furthermore, low testosterone in men with prostate cancer is associated with higher stage and risk of extraprostatic involvement (11). This finding is troubling in light of the high number of patients who do not have a DRE or serum PSA measured prior to starting therapy (5). Prior to TRT this is a higher risk population and if cancer is not discovered before therapy, the testosterone will likely be blamed when the cancer is subsequently discovered, likely at a high stage and grade.

There were 14 men treated with TRT for less than 2 years who had a biopsy for clinical indication, and all these biopsies were negative for cancer, despite several patients having PSA levels jump by 100 to 250%. Clinical trials of TRT typically have reported no increase in mean PSA over time (6) but the mean can hide individual variation. Coward et al. showed PSA to be stable at 1 year intervals up to 5 years and the earliest patient found to have prostate cancer had been treated for 22 months (1). Gerstenbluth et al. found 6 of 54 patients on TRT whose PSA elevated above 4.0ng/mL and 1 patient had a positive biopsy for prostate cancer (7). The reason for this benign jump in PSA in unclear. Indeed one study showed that over 6 months of TRT, there was little change in prostatic testosterone levels or gene expression (12). None of our patients was symptomatic and they did not have category IV prostatitis (13) on their biopsies in proportions that differ from our typical biopsied patients. Based on these limited findings, we wouldn't recommend against a biopsy in a TRT patient with early PSA rise however they may be candidates for other tests that could stratify their risk (eg. PCA3, multiparametric magnetic resonance imaging (14).

There were 21 men on TRT for longer than 2 years who were biopsied and 5 had cancer, although none were greater than Gleason 7. Other long term studies have found men on TRT to develop prostate cancer at a rate similar to those without therapy (15). Furthermore, men who develop prostate cancer while receiving TRT do not appear to have worse outcomes (16). This does emphasize the necessity to follow treatment guidelines and continue age appropriate prostate cancer screening when on TRT.

The primary limitations of this study include: its retrospective nature, lack of exact standardization for timing of tests and interventions, short term follow-up and the relatively small numbers in each group.

In conclusion, we examined the results of prostate biopsy in patients with low testosterone before or after TRT. Patients prior to TRT were at significant risk for aggressive prostate cancer, emphasizing the need for age appropriate screening prior to initiating therapy. None of the men on TRT with an early PSA rise had prostate cancer but prostate cancer did develop in some men after 2 years of therapy. This further supports the majority of evidence that 1 TRT does not cause or promote prostate cancer and 2 safe use of TRT requires regular monitoring with PSA and DRE (when age appropriate).
